# The prevalence and clinical characteristics of nonradiographic axial spondyloarthritis among patients with inflammatory back pain in rheumatology practices: a multinational, multicenter study

**DOI:** 10.1186/s13075-016-1027-9

**Published:** 2016-06-07

**Authors:** Ruben Burgos-Varga, James Cheng-Chung Wei, Mahboob U. Rahman, Nurullah Akkoc, Syed Atiqul Haq, Mohammed Hammoudeh, Ehab Mahgoub, Ena Singh, Lyndon John Llamado, Khalid Shirazy, Sameer Kotak, Constance Hammond, Ron Pedersen, Qi Shen, Bonnie Vlahos

**Affiliations:** Department of Rheumatology, General Hospital of Mexico, Mexico City, Mexico; Division of Allergy, Immunology and Rheumatology, Chung Shan Medical University Hospital, No. 110, Sec. 1, Jianguo N. Road, South District, Taichung City, 40201 Taiwan; Institute of Medicine, Chung Shan Medical University, Taichung City, Taiwan; Graduate Institute of Integrated Medicine, China Medical University, Taichung City, Taiwan; University of Pennsylvania, Philadelphia, PA USA; Pfizer, Collegeville, PA USA; Dokuz Eylül University, İzmir, Turkey; Bangabandhu Sheikh Mujib Medical University, Dhaka, Bangladesh; Hamad Hospital, Doha, Qatar; Pfizer Asia Pacific Region, Makati, Philippines; Pfizer Africa and Middle East Region, Dubai, UAE; Pfizer, New York, NY USA

**Keywords:** Nonradiographic axial SpA, Ankylosing spondylitis, Prevalence, Inflammatory low back pain, Chronic low back pain

## Abstract

**Background:**

Patients with ankylosing spondylitis (AS), who by definition have radiographic sacroiliitis, typically experience symptoms for a decade or more before being diagnosed. Yet, even patients without radiographic sacroiliitis (i.e., nonradiographic axial spondyloarthritis [nr-axSpA]) report a significant disease burden. The primary objective of this study was to estimate the prevalence and clinical characteristics of nr-axSpA among patients with inflammatory back pain (IBP) in rheumatology clinics in a number of countries across the world. A secondary objective was to estimate the prevalence of IBP among patients with chronic low back pain (CLBP).

**Methods:**

Data were collected from 51 rheumatology outpatient clinics in 19 countries in Latin America, Africa, Europe, and Asia. As consecutive patients with CLBP (*N* = 2517) were seen by physicians at the sites, their clinical histories were evaluated to determine whether they met the new Assessment of SpondyloArthritis international Society criteria for IBP. For those who did, their available clinical history (e.g., family history, C-reactive protein [CRP] levels) was documented in a case report form to establish whether they met criteria for nr-axSpA, AS, or other IBP. Patients diagnosed with nr-axSpA or AS completed patient-reported outcome measures to assess disease activity and functional limitations.

**Results:**

A total of 2517 patients with CLBP were identified across all sites. Of these, 974 (38.70 %) fulfilled the criteria for IBP. Among IBP patients, 29.10 % met criteria for nr-axSpA, and 53.72 % met criteria for AS. The prevalence of nr-axSpA varied significantly by region (*p* < 0.05), with the highest prevalence reported in Asia (36.46 %) and the lowest reported in Africa (16.02 %). Patients with nr-axSpA reported mean ± SD Ankylosing Spondylitis Disease Activity Scores based on erythrocyte sedimentation rate and CRP of 2.62 ± 1.17 and 2.52 ± 1.21, respectively, indicating high levels of disease activity (patients with AS reported corresponding scores of 2.97 ± 1.13 and 2.93 ± 1.18). Similarly, the overall Bath Ankylosing Spondylitis Disease Activity Index score of 4.03 ± 2.23 for patients with nr-axSpA (4.56 ± 2.17 for patients with AS) suggested suboptimal disease control.

**Conclusions:**

These results suggest that, in the centers that participated in the study, 29 % of patients with IBP met the criteria for nr-axSpA and 39 % of patients with CLBP had IBP. The disease burden in nr-axSpA is substantial and similar to that of AS, with both groups of patients experiencing inadequate disease control. These findings suggest the need for early detection of nr-axSpA and initiation of available treatment options to slow disease progression and improve patient well-being.

## Background

Spondyloarthritis (SpA) is a constellation of chronic inflammatory conditions that includes ankylosing spondylitis (AS), reactive arthritis, enteropathic arthritis, and psoriatic arthritis, among others [1]. Collectively, the prevalence of SpA varies between 0.5 % and 2 %, making it approximately as common as rheumatoid arthritis [1]. On the basis of their clinical presentation, patients with SpA can be categorized as having axial SpA, in which the spine is predominantly affected, or peripheral SpA, in which the extremities are predominantly affected [2, 3]. According to the 2009 criteria of the Assessment of SpondyloArthritis international Society (ASAS), axial SpA is further categorized into nonradiographic axial SpA (nr-axSpA) and AS, in which the major distinguishing feature is the presence (for AS) or absence (for nr-axSpA) of radiographic sacroiliitis [2, 3].

Approximately 10 % of patients with nr-axSpA develop AS within 2 years and 60 % develop AS within 10 years [4]. Although patients with nr-axSpA may have inflammation detectable by magnetic resonance imaging [5, 6], early detection of axial SpA poses a major challenge to many physicians [2, 3]. Indeed, patients may experience symptoms for a decade or more before receiving a diagnosis [1]. Patients with axial SpA also report a number of impairments in physical functioning and spinal mobility, experience high rates of disability, and contribute to high societal costs [4]. Timely identification of axial SpA may potentially lead to earlier and more effective intervention to delay disease progression [5].

The majority of axial SpA studies have been conducted in Europe, the United States, and Mexico, with information extremely limited in the emerging countries of Latin America, Africa, Central and Eastern Europe, and Asia. The general population prevalence of axial SpA in Europe has been estimated to be between 0.08 % (France) and 0.49 % (Turkey) [6]. The prevalence is slightly higher in the Americas, with researchers in Mexico and the United States reporting rates of 0.60 % and 0.90–1.40 %, respectively [6, 7].

Many of the studies cited above have been focused on AS or axial SpA, leading to a lack of epidemiological and clinical data specific to nr-axSpA. Indeed, even the proportion of axial SpA patients with nr-axSpA is unknown, as estimates vary considerably, ranging from 23 % to 80 % in a recent review of patients with axial SpA [4]. In part, this is due to different methods of assessment and the fact that these studies were not specifically designed to follow patients with undifferentiated SpA and nr-axSpA [4].

The lack of data on nr-axSpA and AS is even more pronounced in emerging countries in Latin America, Europe, Africa, and Asia. The primary objective of this study was to provide prevalence estimates on the presence of nr-axSpA among patients with inflammatory back pain (IBP) in rheumatology clinics across a number of emerging countries. In this study, we also sought to describe the clinical characteristics associated with both nr-axSpA and AS. A secondary objective was to estimate the prevalence of IBP among patients with chronic low back pain (CLBP), given that IBP represents an increasingly important part of identifying patients with axial SpA [2, 3].

## Methods

### Study design

A noninterventional, cross-sectional study was conducted to estimate the prevalence of nr-axSpA among patients with IBP in 51 rheumatology outpatient clinics from 19 countries in Latin America (Colombia, Costa Rica, Mexico, and Peru), Europe inclusive of western Asia (Hungary, Israel, Kazakhstan, Poland, Romania, Russia, and Turkey), Africa (Algeria, Morocco, and South Africa), and Asia focused on southern and eastern Asia (Bangladesh, China, India, Malaysia, and Taiwan). Patient recruitment took place from January through December 2014. The protocol and study materials received institutional review board approval at each participating site; the specific sites are listed in the Acknowledgements section.

Consecutive patients with CLBP were seen by participating physicians at the study sites and were evaluated clinically to determine whether they met the criteria for IBP. Patients who met 2009 ASAS criteria for IBP had their clinical histories further evaluated to determine their eligibility for the medical record abstraction portion of this study. The inclusion criteria were age ≥18 years, CLBP ≥3 months, and four of five of the following parameters: age of onset <40 years, insidious onset, improvement with exercise, no improvement with rest, and pain at night. The exclusion criteria were noninflammatory back pain, a condition that could mimic IBP (e.g., fibromyalgia), the presence of a neuropathic component, unexplained weight loss of >10 kg within the past 6 months, persistent fever, urinary incontinence or retention, saddle anesthesia, decreased anal sphincter tone or fecal incontinence, bilateral lower extremity weakness or numbness, or progressive neurologic deficit.

**Fig. 1:**
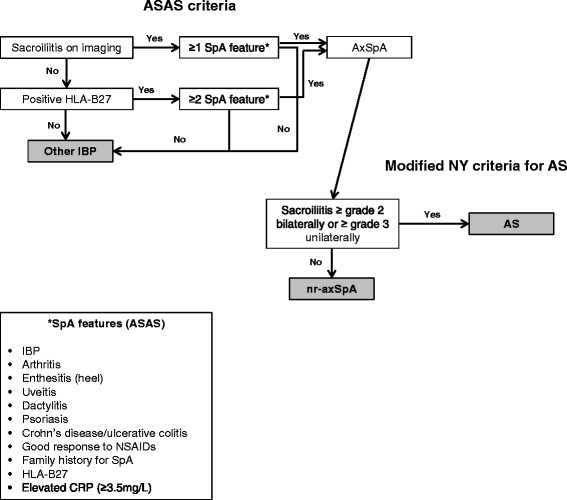
Summary of inflammatory back pain (IBP) group classifications. *AS* ankylosing spondylitis, *ASAS* Assessment of SpondyloArthritis international Society, *axSpA* axial spondyloarthritis, *CRP* C-reactive protein, *HLA-B27* human leukocyte antigen B27, *Modified NY criteria* Modified New York criteria for ankylosing spondylitis, *nr-axSpA* nonradiographic axial spondyloarthritis, *NSAID* nonsteroidal anti-inflammatory drug, *SpA* spondyloarthritis

For patients who met the appropriate inclusion criteria and provided written informed consent, a medical record abstraction using a case report form (CRF) was performed to determine whether the patients met the criteria for AS, nr-axSpA, or other forms of IBP (“other IBP” hereafter) based on ASAS criteria for axial SpA and the Modified New York (Modified NY) criteria for AS (Fig. 1). More specifically, patients who did not meet ASAS criteria were classified as having other IBP. Patients who met ASAS criteria for axial SpA but did not meet Modified NY criteria were classified as having nr-axSpA. Patients who met both ASAS criteria for axial SpA and Modified NY criteria were classified as having AS.

Patients who were diagnosed with AS or nr-axSpA were provided with a brief survey to evaluate their patient-reported outcomes (PROs). It is important to note that *diagnosed* refers to the physician’s classification of that patient as indicated in the medical record. It is not known what information was used to make this assessment. The classification of patients based on ASAS criteria for axial SpA and Modified NY criteria for AS, as described above, was an analytical exercise performed by the authors after data collection; it was not conducted in real time as patients were enrolled into the study. As a result, the study depended upon the physician’s classification (referred hereafter as *diagnosis* to distinguish the methods) in order to identify patients eligible to receive the survey, even if this differed from our methods of classification using the ASAS criteria for axial SpA and the Modified NY criteria for AS. This survey was completed entirely by the patient in the waiting room to avoid any influence from the investigator. At the end of subject recruitment, all completed materials were collected on-site and checked for completion, with the exception of the patient questionnaire, which remained confidential.

### Measures

The CRF assessed information on each patient’s demographics (age, sex, race/ethnicity), general health history (body mass index [BMI], years of experienced CLBP), and disease history (human leukocyte antigen B27 [HLA-B27] test results, C-reactive protein [CRP] results, erythrocyte sedimentation rate [ESR] results, family history, nonsteroidal anti-inflammatory drug [NSAID] response). The patient survey included the Ankylosing Spondylitis Disease Activity Score (ASDAS; both ESR and CRP versions), Bath Ankylosing Spondylitis Disease Activity Index (BASDAI), Bath Ankylosing Spondylitis Functional Index (BASFI), and Bath Ankylosing Spondylitis Metrology Index (BASMI).

### Statistical analysis

The analysis of the primary objectives (prevalence and clinical characteristics of nr-axSpA) was focused on patients with IBP who met the inclusion or exclusion criteria described above. The analysis of the secondary objective (prevalence of IBP among patients with CLBP) was focused on all patients with CLBP. Frequencies, percentages, and 95 % CIs were reported for binary and/or categorical variables. Counts, means, and SDs were reported for continuous variables. Statistical differences across geographical regions were analyzed using chi-square tests and one-way analysis of variance for categorical and continuous variables, respectively.

## Results

### Prevalence of nr-axSpA overall, by region, and by sex

**Fig. 2:**
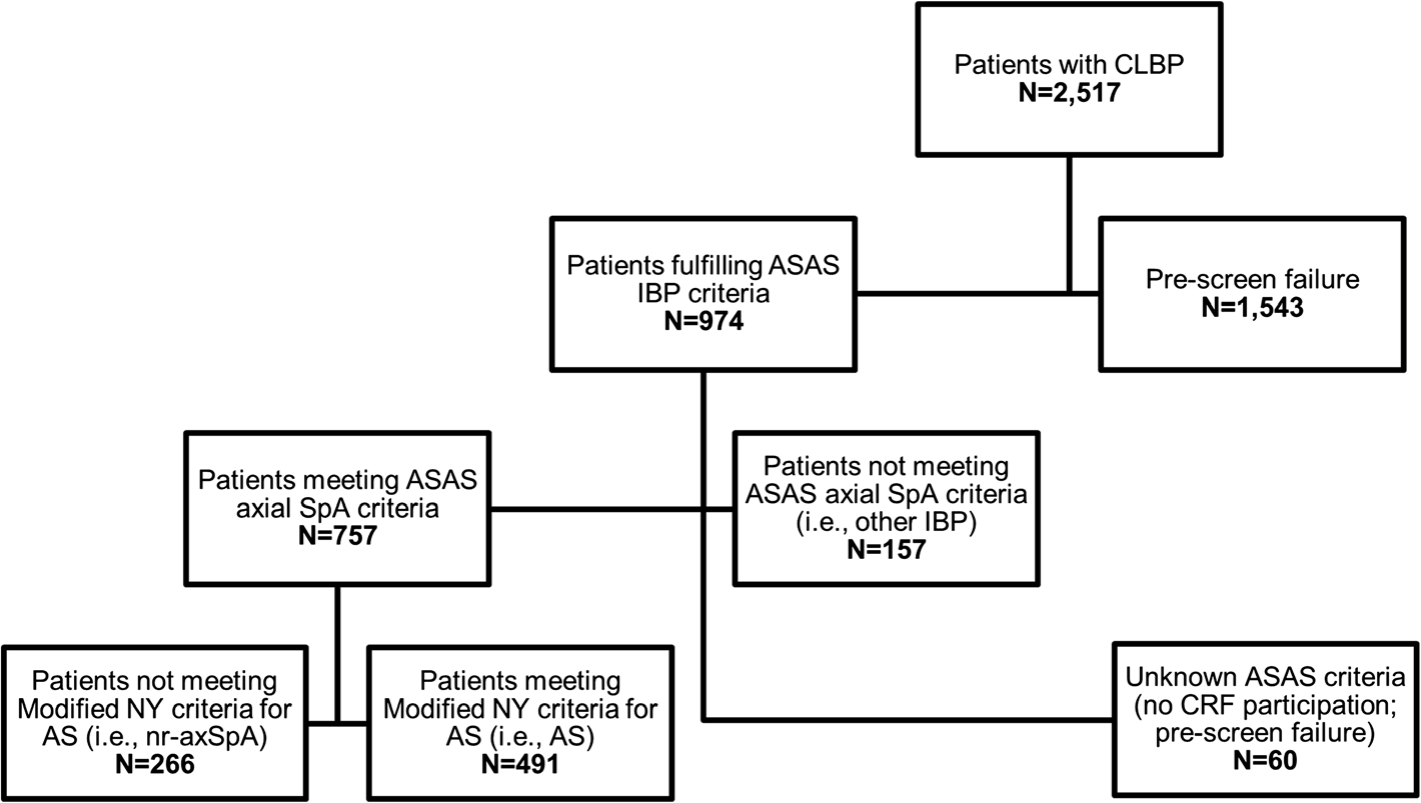
Study flowchart. *AS* ankylosing spondylitis, *ASAS* Assessment of SpondyloArthritis international Society, *CLBP* chronic low back pain, *CRF* case report form, *IBP* inflammatory back pain, *Modified NY criteria* Modified New York criteria for ankylosing spondylitis, *nr-axSpA* nonradiographic axial spondyloarthritis, SpA spondyloarthritis

A total of 2517 patients with CLBP were identified across all sites (Fig. 2). Of these, 974 (38.70 %) fulfilled the criteria for IBP and were advanced to the CRF portion of the study for assessment of IBP group status (i.e., nr-axSpA, AS, or other IBP). Overall, 29.10 % (95 % CI 26.15–32.05 %) of patients with IBP met the criteria for nr-axSpA (Table 1). The prevalence of AS among patients with IBP was 53.72 % (95 % CI 50.48–56.96 %). The prevalence of nr-axSpA varied significantly by region (*p* < 0.05), with the highest prevalence reported in Asia (36.46 %, 95 % CI 31.64–41.28 %) and the lowest reported in Africa (16.02 %, 95 % CI 11.00–21.04 %). The prevalence of nr-axSpA was similar among males (28.74 %, 95 % CI 25.07–32.41 %) and females (29.75 %, 95 % CI 24.77–34.74 %) with IBP.

**Table 1:** Prevalence of nonradiographic axial spondyloarthritis among patients with inflammatory back pain across geographic regions

	Total	Latin America	Africa	Europe	Asia	*p* Value
Overall sample						
Number of patients	914	26	206	298	384	
nr-axSpA, *n* (%)	266 (29.10 %)	5 (19.23 %)	33 (16.02 %)	88 (29.53 %)	140 (36.46 %)	<0.001
95 % CI	(26.15–32.05)	(4.05–34.41)	(11.00–21.04)	(24.34–34.72)	(31.64–41.28)	
Among males						
Number of patients	588	12	116	204	256	
nr-axSpA, *n* (%)	169 (28.74 %)	4 (33.33 %)	18 (15.52 %)	57 (27.94 %)	90 (35.16 %)	<0.001
95 % CI	(25.07–32.41)	(6.58–60.08)	(8.91–22.13)	(21.77–34.12)	(29.29–41.02)	
Among females						
Number of patients	326	14	90	94	128	
nr-axSpA, *n* (%)	97 (29.75 %)	1 (7.14 %)	15 (16.67 %)	31 (32.98 %)	50 (39.06 %)	<0.001
95 % CI	(24.77–34.74)	(0.00–20.70)	(8.93–24.41)	(23.42–42.53)	(30.57–47.56)	

*nr-axSpA* nonradiographic axial spondyloarthritis

The *p* values represent the omnibus statistical comparison of percentages across geographic regions

### Demographic characteristics of patients with nr-axSpA

Patients with nr-axSpA had a mean age of 34.75 years (SD 10.03); 36.47 % were female, and 55.64 % were white (Table 2). These figures contrasted with patients with AS, who had a mean age of 39.03 years (SD 11.38); 28.72 % were female, and 68.23 % were white. Minimal regional differences were observed with respect to the demographic characteristics of patients with nr-axSpA (Table 3). Patients with nr-axSpA were oldest in Africa (36.67 years) and youngest in Asia (33.24 years) (*p* < 0.05); however, no differences in sex, BMI, years since CLBP presentation, or age of IBP onset were observed across regions.

**Table 2:** Demographic characteristics of nonradiographic axial spondyloarthritis, ankylosing spondylitis, and other inflammatory back pain patients

	Total (*N* = 914)	nr-axSpA (*n* = 266)	AS (*n* = 491)	Other IBP (*n* = 157)	*p* Value
Sex					<0.001
Male, *n* (%)	588 (64.33 %)	169 (63.53 %)	350 (71.28 %)	69 (43.95 %)	
95 % CI	(61.22–67.44)	(57.74–69.33)	(67.27–75.29)	(36.17–51.73)	
Female, *n* (%)	326 (35.67 %)	97 (36.47 %)	141 (28.72 %)	88 (56.05 %)	
95 % CI	(32.56–38.78)	(30.67–42.26)	(24.71–32.73)	(48.27–63.83)	
Sex					<0.001
Male, *n* (row %)	588 (100.00 %)	169 (28.75 %)	350 (59.52 %)	69 (11.73 %)	
95 % CI		(25.08–32.41)	(55.54–63.50)	(9.13–14.34)	
Female, *n* (row %)	326 (100.00 %)	97 (29.75 %)	141 (43.25 %)	88 (26.99 %)	
95 % CI		(24.78–34.73)	(37.86–48.64)	(22.17–31.82)	
Age, years, mean ± SD	38.68 ± 12.02	34.75 ± 10.03	39.03 ± 11.38	44.26 ± 14.48	<0.001
Race/ethnicity					<0.001
White, *n* (%)	596 (65.21 %)	148 (55.64 %)	335 (68.23 %)	113 (71.97 %)	
95 % CI	(62.11–68.30)	(49.66–61.62)	(64.10–72.35)	(64.94–79.01)	
Asian, *n* (%)	116 (12.69 %)	55 (20.68 %)	55 (11.20 %)	6 (3.82 %)	
95 % CI	(10.53–14.85)	(15.80–25.55)	(8.41–14.00)	(0.82–6.83)	
Black, *n* (%)	12 (1.31 %)	2 (0.75 %)	2 (0.41 %)	8 (5.10 %)	
95 % CI	(0.57–2.05)	(0.00–1.79)	(0.00–0.97)	(1.65–8.54)	
Unknown, *n* (%)	190 (20.79 %)	61 (22.93 %)	99 (20.16 %)	30 (19.11 %)	
95 % CI	(18.15–23.42)	(17.87–27.99)	(16.61–23.72)	(12.95–25.27)	
BMI category					0.024
Underweight, *n* (%)	55 (6.02 %)	12 (4.51 %)	38 (7.74 %)	5 (3.18 %)	
95 % CI	(4.47–7.56)	(2.01–7.01)	(5.37–10.11)	(0.43–5.94)	
Normal weight, *n* (%)	455 (49.78 %)	150 (56.39 %)	236 (48.07 %)	69 (43.95 %)	
95 % CI	(46.53–53.03)	(50.42–62.36)	(43.64–52.49)	(36.17–51.73)	
Overweight, *n* (%)	279 (30.53 %)	78 (29.32 %)	146 (29.74 %)	55 (35.03 %)	
95 % CI	(27.53–33.52)	(23.84–34.8)	(25.68–33.79)	(27.56–42.51)	
Obese, *n* (%)	121 (13.24 %)	24 (9.02 %)	70 (14.26 %)	27 (17.20 %)	
95 % CI	(11.04–15.44)	(5.57–12.47)	(11.16–17.36)	(11.28–23.11)	
Unknown, *n* (%)	4 (0.44 %)	2 (0.75 %)	1 (0.20 %)	1 (0.64 %)	
95 % CI	(0.01–0.87)	(0.00–1.79)	(0.00–0.60)	(0.00–1.88)	
Years since CLBP presentation					<0.001
Number of patients	808	232	435	141	
Mean ± SD, years	9.09 ± 9.07	6.32 ± 7.61	10.91 ± 9.37	8.05 ± 9.10	
Age of IBP onset					<0.001
Number of patients	908	264	488	156	
Mean ± SD, years	28.8 ± 9.7	27.8 ± 7.3	27.0 ± 7.7	36.2 ± 14.2	

*Abbreviations: AS* ankylosing spondylitis, *BMI* body mass index, *CLBP* chronic low back pain, *CRF* case report form, *IBP* inflammatory back pain, *nr-axSpA* nonradiographic axial spondyloarthritis

Broad race categories (white vs. black vs. Asian vs. unknown) were created on the basis of physician-reported patient ethnicity: white = Indian, Indo-Aryan, Dravidian, Bengali, Arab, Iranian, white, Jewish, Azeri, Mestizo, Amerindian, Berber; black = black, mulatto, mulato, zambo, black African, colored; Asian = Han Chinese, non-Han Chinese, Taiwanese, Chinese, aborigine, Malay, indigenous, mongoloid, Asian; unknown = other or missing

The *p* values represent the omnibus statistical comparison of percentages (or means) across IBP groups based on the chi-square (or *F*-test) values

“Other IBP” refers to patients who did not meet the Assessment of SpondyloArthritis international Society classification criteria for axial spondyloarthritis

**Table 3:** Demographic characteristics of patients with nonradiographic axial spondyloarthritis across geographic regions

	Africa (*n* = 33)	Europe (*n* = 88)	Asia (*n* = 140)	*p* Value
Sex				0.609
Male, *n* (%)	18 (54.55 %)	57 (64.77 %)	90 (64.29 %)	
95 % CI	(37.45–71.64)	(54.73–74.82)	(56.30–72.27)	
Female (%)	15 (45.45 %)	31 (35.23 %)	50 (35.71 %)	
95 % CI	(28.36–62.55)	(25.18–45.27)	(27.73–43.70)	
Age, years mean ± SD	36.67 ± 10.73	36.02 ± 10.56	33.24 ± 9.26	0.035
BMI category				0.21
Underweight, *n* (%)	1 (3.03 %)	4 (4.55 %)	7 (5.00 %)	
95 % CI	(0.00–8.92)	(0.17–8.93)	(1.37–8.63)	
Normal weight, *n* (%)	16 (48.48 %)	51 (57.95 %)	81 (57.86 %)	
95 % CI	(31.32–65.65)	(47.57–68.34)	(49.62–66.09)	
Overweight, *n* (%)	13 (39.39 %)	19 (21.59 %)	44 (31.43 %)	
95 % CI	(22.61–56.17)	(12.94–30.24)	(23.69–39.17)	
Obese, *n* (%)	2 (6.06 %)	14 (15.91 %)	7 (5.00 %)	
95 % CI	(0.00–14.25)	(8.22–23.6)	(1.37–8.63)	
Unknown, *n* (%)	1 (3.03 %)	0 (0.00 %)	1 (0.71 %)	
95 % CI	(0.00–8.92)	–	(0.00–2.12)	
Years since CLBP presentation				0.462
Number of patients	29	74	124	
Mean ± SD, years	8.02 ± 10.01	6.68 ± 9.33	5.67 ± 5.70	
Age of IBP onset				0.115
Number of patients	33	87	139	
Mean ± SD, years	28.4 ± 8.7	28.3 ± 6.5	27.1 ± 7.3	

*BMI* body mass index, *CLBP* chronic low back pain

The *p* values represent the omnibus statistical comparison of percentages (or means) across regions based on the chi-square (or *F*-test) values

### Clinical characteristics of patients with nr-axSpA

Patients with nr-axSpA experienced CLBP for 6.32 ± 7.61 years (compared with 10.91 years for patients with AS) (Table 2). HLA-B27 test results were available for 71.05 % of those with nr-axSpA, and 82.78 % of them had a positive test result (Table 4). Proportions of 68.14 % and 37.30 % of patients with nr-axSpA had elevated CRP and ESR values, respectively. For context, proportions of 77.20 % and 48.92 % of patients with AS had elevated CRP and ESR values, respectively.

**Table 4:** Clinical characteristics and HLA-B27 test results of patients with nonradiographic axial spondyloarthritis, ankylosing spondylitis, and other inflammatory back pain

	Total (*N* = 914)	nr-axSpA (*n* = 266)	AS (*n* = 491)	Other IBP (*n* = 157)	*p* Value
HLA-B27 test results (when available)					<0.001
Positive, *n* (%)	397 (72.98 %)	135 (71.43 %)	250 (82.78 %)	12 (22.64 %)	
Negative, *n* (%)	147 (27.02 %)	54 (28.57 %)	52 (17.22 %)	41 (77.36 %)	
Years from IBP to SpA diagnosis					0.747
Number of patients	179	98	27	54	
Mean ± SD, years	5.40 ± 7.98	5.21 ± 7.69	6.48 ± 8.53	5.19 ± 8.32	
Family history of SpA					<0.001
Yes, *n* (%)	220 (24.07 %)	70 (26.32 %)	132 (26.88 %)	18 (11.46 %)	
No, *n* (%)	650 (71.12 %)	180 (67.67 %)	343 (69.86 %)	127 (80.89 %)	
Results not available, *n* (%)	44 (4.81 %)	16 (6.02 %)	16 (3.26 %)	12 (7.64 %)	
Family history of SpA (excluding missing)					<0.001
Yes, *n* (%)	220 (25.29 %)	70 (28.00 %)	132 (27.79 %)	18 (12.41 %)	
No, *n* (%)	650 (74.71 %)	180 (72.00 %)	343 (72.21 %)	127 (87.59 %)	
NSAID response					0.125
Positive, *n* (%)	657 (71.88 %)	183 (68.80 %)	358 (72.91 %)	116 (73.89 %)	
Negative, *n* (%)	231 (25.27 %)	72 (27.07 %)	125 (25.46 %)	34 (21.66 %)	
Results not available, *n* (%)	26 (2.84 %)	11 (4.14 %)	8 (1.63 %)	7 (4.46 %)	
Most recent CRP value					0.146
Number of patients	767	226	421	120	
Mean ± SD, mg/L	16.7 ± 26.0	15.8 ± 28.2	18.2 ± 25.3	13.2 ± 23.5	
Most recent CRP level					<0.001
Not elevated (<3.5 mg/L), *n* (%)	219 (23.96 %)	72 (27.07 %)	96 (19.55 %)	51 (32.48 %)	
Elevated (≥3.5 mg/L), *n* (%)	548 (59.96 %)	154 (57.89 %)	325 (66.19 %)	69 (43.95 %)	
Results not available, *n* (%)	147 (16.08 %)	40 (15.04 %)	70 (14.26 %)	37 (23.57 %)	
Most recent CRP level (excluding missing)					<0.001
Not elevated (<3.5 mg/L), *n* (%)	219 (28.55 %)	72 (31.86 %)	96 (22.80 %)	51 (42.50 %)	
Elevated (≥3.5 mg/L), *n* (%)	548 (71.45 %)	154 (68.14 %)	325 (77.20 %)	69 (57.50 %)	
Most recent ESR value					0.009
Number of patients	846	252	464	130	
Mean ± SD, mm/h	29.0 ± 23.6	26.8 ± 23.9	31.2 ± 23.6	25.3 ± 22.8	
Most recent ESR level					<0.001
Not elevated (<28 mm/h), *n* (%)	482 (52.74 %)	158 (59.40 %)	237 (48.27 %)	87 (55.41 %)	
Elevated (≥28 mm/h), *n* (%)	364 (39.82 %)	94 (35.34 %)	227 (46.23 %)	43 (27.39 %)	
Results not available, *n* (%)	68 (7.44 %)	14 (5.26 %)	27 (5.50 %)	27 (17.20 %)	
Most recent ESR level (excluding missing)					<0.001
Not elevated (<28 mm/h), *n* (%)	482 (56.97 %)	158 (62.70 %)	237 (51.08 %)	87 (66.92 %)	
Elevated (≥28 mm/h), *n* (%)	364 (43.03 %)	94 (37.30 %)	227 (48.92 %)	43 (33.08 %)	

*Abbreviations: AS* ankylosing spondylitis, *CRP* C-reactive protein, *ESR* erythrocyte sedimentation rate, *HLA-B27* human leukocyte antigen B27, *IBP* inflammatory back pain, *nr-axSpA* nonradiographic axial spondyloarthritis, *NSAID* nonsteroidal anti-inflammatory drug, *SpA* spondyloarthritis

The *p* values represent the omnibus statistical comparison of percentages (or means) across IBP groups based on the chi-square values

Several clinical characteristics varied across regions among those with nr-axSpA (Table 5). Patients in Europe were the most likely to have a positive HLA-B27 test result (84.85 %), and patients in Asia were the least likely (62.24 %) (*p* < 0.05). Patients in Asia were the most likely to have an elevated ESR value (48.89 %), and patients in Europe were the least likely (20.48 %) (*p* < 0.05). No differences in family history or NSAID response were observed.

**Table 5:** Clinical characteristics and HLA-B27 test results of patients with nonradiographic axial spondyloarthritis across geographic regions

	Africa (*n* = 33)	Europe (*n* = 88)	Asia (*n* = 140)	*p* Value
Laboratory-confirmed SpA (HLA-B27)				0.062
Positive, *n* (%)	15 (45.45 %)	56 (63.64 %)	61 (43.57 %)	
Negative, *n* (%)	6 (18.18 %)	10 (11.36 %)	37 (26.43 %)	
Results not available, *n* (%)	12 (36.36 %)	22 (25.00 %)	42 (30.00 %)	
Laboratory-confirmed SpA (excluding missing) (HLA-B27)				0.019
Positive, *n* (%)	15 (71.43 %)	56 (84.85 %)	61 (62.24 %)	
Negative, *n* (%)	6 (28.57 %)	10 (15.15 %)	37 (37.76 %)	
Family history of SpA				0.231
Yes, *n* (%)	9 (27.27 %)	18 (20.45 %)	43 (30.71 %)	
No, *n* (%)	22 (66.67 %)	67 (76.14 %)	86 (61.43 %)	
Results not available, *n* (%)	2 (6.06 %)	3 (3.41 %)	11 (7.86 %)	
Family history of SpA (excluding missing)				0.125
Yes, *n* (%)	9 (29.03 %)	18 (21.18 %)	43 (33.33 %)	
No, *n* (%)	22 (70.97 %)	67 (78.82 %)	86 (66.67 %)	
NSAID response				0.264
Positive, *n* (%)	22 (66.67 %)	64 (72.73 %)	93 (66.43 %)	
Negative, *n* (%)	7 (21.21 %)	22 (25.00 %)	42 (30.00 %)	
Results not available, *n* (%)	4 (12.12 %)	2 (2.27 %)	5 (3.57 %)	
Most recent CRP level				0.214
Not elevated (<3.5 mg/L), *n* (%)	10 (30.30 %)	21 (23.86 %)	37 (26.43 %)	
Elevated (≥3.5 mg/L), *n* (%)	18 (54.55 %)	55 (62.50 %)	80 (57.14 %)	
Results not available, *n* (%)	5 (15.15 %)	12 (13.64 %)	23 (16.43 %)	
Most recent CRP level (excluding missing)				0.104
Not elevated (<3.5 mg/L), *n* (%)	10 (35.71 %)	21 (27.63 %)	37 (31.62 %)	
Elevated (≥3.5 mg/L), *n* (%)	18 (64.29 %)	55 (72.37 %)	80 (68.38 %)	
Most recent ESR level				<0.001
Not elevated (<28 mm/h), *n* (%)	20 (60.61 %)	66 (75.00 %)	69 (49.29 %)	
Elevated (≥28 mm/h), *n* (%)	9 (27.27 %)	17 (19.32 %)	66 (47.14 %)	
Results not available, *n* (%)	4 (12.12 %)	5 (5.68 %)	5 (3.57 %)	
Most recent ESR level (excluding missing)				<0.001
Not elevated (<28 mm/h), *n* (%)	20 (68.97 %)	66 (79.52 %)	69 (51.11 %)	
Elevated (≥28 mm/h), *n* (%)	9 (31.03 %)	17 (20.48 %)	66 (48.89 %)	

*Abbreviations: CRP* C-reactive protein, *ESR* erythrocyte sedimentation rate, *HLA-B27* human leukocyte antigen B27, *NSAID* nonsteroidal anti-inflammatory drug, *SpA* spondyloarthritis

The *p* values represent the omnibus statistical comparison of percentages (or means) across regions based on the chi-square values

### Delay from IBP and CLBP to nr-axSpA or AS diagnosis

For patients who received a diagnosis of nr-axSpA, there was a mean delay of 5.21 ± 7.69 years between the presentation of IBP and diagnosis (Table 4). There was a mean delay of 6.48 ± 8.53 years between the presentation of IBP and diagnosis for patients with AS.

### Patient-reported outcomes in nr-axSpA and AS

The mean disease activity levels for patients with nr-axSpA were 2.62 ± 1.17 and 2.52 ± 1.21 for the ESR and CRP versions of the ASDAS, respectively, suggesting a high level of disease activity (i.e., ≥2.1) (Table 6). The mean overall BASDAI score was 4.03 ± 2.32 for patients with nr-axSpA, indicating a suboptimal level of disease control. Finally, BASFI score (3.20 ± 2.47) and BASMI scores (11-point version 2.41 ± 1.54, 3-point version 1.62 ± 1.51, linear function version 3.71 ± 2.77) indicated a significant burden for patients with nr-axSpA and were relatively comparable to BASFI score (4.09 ± 2.59) and BASMI scores (11-point version 4.09 ± 2.06, 3-point version 3.40 ± 2.25, linear function version 4.77 ± 2.38) for patients with AS. No differences in PRO measures were observed across regions, with the exception of the 3-point BASMI version (Africa = 2.59 ± 1.52, Europe = 1.46 ± 1.53, Asia = 1.30 ± 1.36; *p* < 0.05).

**Table 6:** Clinical outcomes and patient-reported outcome measures for patients with nonradiographic axial spondyloarthritis, ankylosing spondylitis, and other inflammatory back pain

	Total (*N* = 686)	nr-axSpA (*N* = 188)	AS (*N* = 413)	Other IBP (*N* = 85)	*p* Value
ASDAS score (ESR)					0.003
Number of patients	619	167	378	74	
Mean ± SD	2.87 ± 1.14	2.62 ± 1.17	2.97 ± 1.13	2.92 ± 1.01	
ASDAS score (CRP)					0.002
Number of patients	559	149	346	64	
Mean ± SD	2.81 ± 1.19	2.52 ± 1.21	2.93 ± 1.18	2.81 ± 1.17	
BASDAI					0.010
Number of patients	681	186	412	83	
Mean ± SD	4.44 ± 2.24	4.03 ± 2.32	4.56 ± 2.17	4.77 ± 2.29	
BASFI					<0.001
Number of patients	683	187	411	85	
Mean ± SD	4.09 ± 2.59	3.20 ± 2.47	4.43 ± 2.57	4.38 ± 2.49	
BASMI (11-point)					<0.001
Number of patients	575	154	353	68	
Mean ± SD	3.55 ± 2.03	2.41 ± 1.54	4.09 ± 2.06	3.33 ± 1.75	
BASMI (3-point)					<0.001
Number of patients	564	146	347	71	
Mean ± SD	2.83 ± 2.19	1.62 ± 1.51	3.40 ± 2.25	2.52 ± 2.03	
BASMI (linear function)					<0.001
Number of patients	686	188	413	85	
Mean ± SD	4.44 ± 2.55	3.71 ± 2.77	4.77 ± 2.38	4.47 ± 2.50	

*Abbreviation: AS* ankylosing spondylitis, *ASDAS* Ankylosing Spondylitis Disease Activity Score, *BASDAI* Bath Ankylosing Spondylitis Disease Activity Index, *BASFI* Bath Ankylosing Spondylitis Functional Index, *BASMI* Bath Ankylosing Spondylitis Metrology Index, *CRP* C-reactive protein, *ESR* erythrocyte sedimentation rate, *IBP* inflammatory back pain, *nr-axSpA* nonradiographic axial spondyloarthritis

The *p* values represent the omnibus statistical comparison of percentages (or means) across IBP groups based on the *F*-test values

## Discussion

In this study, 39 % of patients referred to rheumatology clinics with CLBP met the ASAS criteria for IBP. Further, 29 % of patients with IBP met the criteria for nr-axSpA. The proportion of axial SpA patients with nr-axSpA was within the range (23–80 %) reported in the literature [4, 8], though on the lower end of prior estimates.

Our data suggest a higher percentage of males among patients with nr-axSpA (64 %) relative to the percentages in other published noninterventional studies (34–50 %) [9, 10] and most clinical trials (48–64 %) [11–16]. There were a number of methodological differences across these studies (e.g., inclusion and exclusion criteria, country), but it is unclear which of these factors would help to explain the differences in results. Further research is necessary.

We found that patients with nr-axSpA were the youngest, and they experienced CLBP for the shortest duration at slightly over 6 years compared with nearly 11 years for patients with AS. Although no age differences were found in past literature reviews between patients with nr-axSpA and those with AS [4, 8], several prior studies have found a longer symptom duration for patients with AS [4–8, 10, 17, 18]. This is to be expected, given that nr-axSpA and AS likely represent a progression in the spectrum of the same disease.

Among patients who had been diagnosed with nr-axSpA, there was a delay of approximately 5 years between presentation of IBP and diagnosis. This finding was consistent with the study by Poddubnyy and colleagues, who which also found a gap of slightly more than 5 years between back pain and the assessment of nr-axSpA [17]. However, it should be noted that many patients who met the criteria for nr-axSpA were not diagnosed even after this period of several years, reinforcing the importance of increasing the awareness of, and adherence to, ASAS classification criteria for timely diagnosis and initiation of treatment.

Among those with HLA-B27 test results, 83 % of the patients with nr-axSpA had positive results. This finding is consistent with what was summarized by Boonen et al. [4] and reported directly in two recent noninterventional studies (73 % in a study by Poddubnyy and colleagues [11] and 86 % in a study by Kiltz and colleagues [18]. Over two-thirds (68 %) of patients with nr-axSpA had elevated CRP values (≥3.5 mg/L), which was nearly as high as the percentage of patients with AS who had elevated CRP values (77 %).

Data derived from the patient surveys demonstrated a high level of disease activity and a suboptimal level of disease control, as assessed using the ASDAS and BASDAI, respectively, for patients with nr-axSpA. It is important to note that even patients with nr-axSpA [19], who are in an earlier phase in the course of axial SpA, exhibited a significant burden that was comparable to that of patients with AS. BASDAI scores in our study were lower than those reported in a review of clinical trial results of biologic treatments [8], suggesting a less severe patient-reported burden in this real-world patient population. The levels of functional impairment (BASFI) and limitations (BASMI) were comparable to those reported for clinical trial populations [4]. Given the early age of onset for nr-axSpA, these impairment data suggest that patients can experience a substantial level of burden for many years. This further illustrates the importance of identifying patients early in order to slow disease progression.

### Limitations

Limitations of this epidemiological study include the use of a single assessment with a questionnaire and CRF that may not adequately capture a comprehensive medical history for a particular patient. It is also important to mention that this was an observational study, so not all patients had complete information available. This could have affected the classification of patients and therefore the prevalence estimates. For example, because a positive HLA-B27 test is one way to classify a patient as having nr-axSpA instead of other IBP, missing HLA-B27 data would underestimate the number of nr-axSpA patients relative to other IBP patients. Another limitation was the lack of available information on the other IBP group. Although this group also had poor outcomes based on the PRO data, the explanation for this finding is unclear without knowing more about the composition of the other IBP group. Patient surveys were administered only to patients who were diagnosed with AS and nr-axSpA, so patients who were not diagnosed with either condition, even if they met the appropriate classification criteria, did not provide PRO data. The external validity of the study is dependent on the extent to which patients at the selected rheumatology practices are representative of all IBP patients in these countries. Because these sites were selected for being major centers for the treatment of SpA, it is possible the patients who are managed by these sites are fundamentally different (e.g., more severe disease).

## Conclusions

The results of the present study suggest that approximately one-third of patients with IBP meet ASAS criteria for nr-axSpA. Patients with nr-axSpA, as compared with patients with AS, tend to be younger and experience symptoms for a shorter time before diagnosis. The PRO data suggest that the overall disease burden in nr-axSpA is substantial and similar to that in AS, with both groups of patients experiencing inadequate disease control. These findings show the continued need for early diagnosis of nr-axSpA across Latin America, Europe, Africa, and Asia. These findings also emphasize the importance of early initiation of available treatment options to slow disease progression and improve patient well-being in these patients’ most productive years of life.

## Abbreviations

AS, ankylosing spondylitis; ASAS, Assessment of SpondyloArthritis international Society; ASDAS, Ankylosing Spondylitis Disease Activity Score; BASDAI, Bath Ankylosing Spondylitis Disease Activity Index; BASFI, Bath Ankylosing Spondylitis Functional Index; BASMI, Bath Ankylosing Spondylitis Metrology Index; BMI, body mass index; CLBP, chronic low back pain; CRF, case report form; CRP, C-reactive protein; ESR, erythrocyte sedimentation rate; HLA-B27, human leukocyte antigen B27; IBP, inflammatory back pain; Modified NY criteria, Modified New York criteria for ankylosing spondylitis; nr-axSpA, nonradiographic axial spondyloarthritis; NSAID, nonsteroidal anti-inflammatory drug; PRO, patient-reported outcome; SpA, spondyloarthritis95 % CI
