# Erratum to: The prevalence and clinical characteristics of nonradiographic axial spondyloarthritis among patients with inflammatory back pain in rheumatology practices: a multinational, multicenter study

**DOI:** 10.1186/s13075-016-1066-2

**Published:** 2016-07-04

**Authors:** Ruben Burgos-Vargas, James Cheng-Chung Wei, Mahboob U. Rahman, Nurullah Akkoc, Syed Atiqul Haq, Mohammed Hammoudeh, Ehab Mahgoub, Ena Singh, Lyndon John Llamado, Khalid Shirazy, Sameer Kotak, Constance Hammond, Ron Pedersen, Qi Shen, Bonnie Vlahos

**Affiliations:** Department of Rheumatology, General Hospital of Mexico, Mexico City, Mexico; Division of Allergy, Immunology and Rheumatology, Chung Shan Medical University Hospital, No. 110, Sec. 1, Jianguo N. Road, Taichung City, 40201 South District Taiwan; Institute of Medicine, Chung Shan Medical University, Taichung City, Taiwan; Graduate Institute of Integrated Medicine, China Medical University, Taichung City, Taiwan; University of Pennsylvania, Philadelphia, PA USA; Pfizer, Collegeville, PA USA; Dokuz Eylül University, İzmir, Turkey; Bangabandhu Sheikh Mujib Medical University, Dhaka, Bangladesh; Hamad Hospital, Doha, Qatar; Pfizer Asia Pacific Region, Makati, Philippines; Pfizer Africa and Middle East Region, Dubai, United Arab Emirates; Pfizer, New York, NY USA

Unfortunately, after publication of this article [1], it was noticed that the name of the first author was incorrect. The corrected author list can be seen above and the original article has been updated to reflect this.
